# Seven-Membered Lactam Derivatives of Podophyllotoxins as New Pesticidal Agents

**DOI:** 10.1038/s41598-017-04136-3

**Published:** 2017-06-20

**Authors:** Xiaoyan Zhi, Yuanyuan Zhang, Jiulin Huang, Hui Xu

**Affiliations:** 1 Research Institute of Pesticidal Design & Synthesis, College of Chemistry and Pharmacy, Northwest A&F University, Yangling, 712100 Shaanxi Province P. R. China; 2 College of Agriculture, Shanxi Agriculture University, Taigu, 030801 Shanxi Province P. R. China; 3 Shaanxi Key Laboratory of Natural Products & Chemical Biology, and College of Plant Protection, Northwest A&F University, Yangling, 712100 Shaanxi Province P. R. China

## Abstract

As a continuation of our efforts to discover and develop natural-product-based insecticidal agents, three novel and unusual 7-membered lactam derivatives of podophyllotoxin were prepared by thionyl chloride-mediated ring-expanded Beckmann rearrangement. The steric configurations of 3a–c were unambiguously identified by X-ray crystallography. It demonstrated that the configuration of the picropodophyllotoxin C4-oximes could also be confirmed by the corresponding C-ring expansion products via Beckmann rearrangement. Moreover, it was obviously further testified that when picropodophyllones reacted with hydroxylamine hydrochloride, only *E* configuration of picropodophyllotoxin C4-oximes was selectively produced. Compounds 3b and 3c showed more potent pesticidal activity than toosendanin against oriental armyworm, *Mythimna separata* (Walker).

## Introduction

Oriental armyworm, *Mythimna separata* Walker, is a typically polyphagous and gluttonous lepidopteran pest, and hard to control. Nowadays, the use of chemical insecticides continues to play an important role in the control of insect pests. However, the repeat and increasing application of those agrochemicals has led to the global dissemination of resistance in insect pests populations, and the serious human health and environmental problems^1–5^. In addition, the discovery and development of new insecticidal agents from plant secondary metabolites, or by using them as the lead compounds for further structural modifications, has recently been received much attention owing to their less or slower resistance development and low toxicity^6–8^.

**Figure 1:**
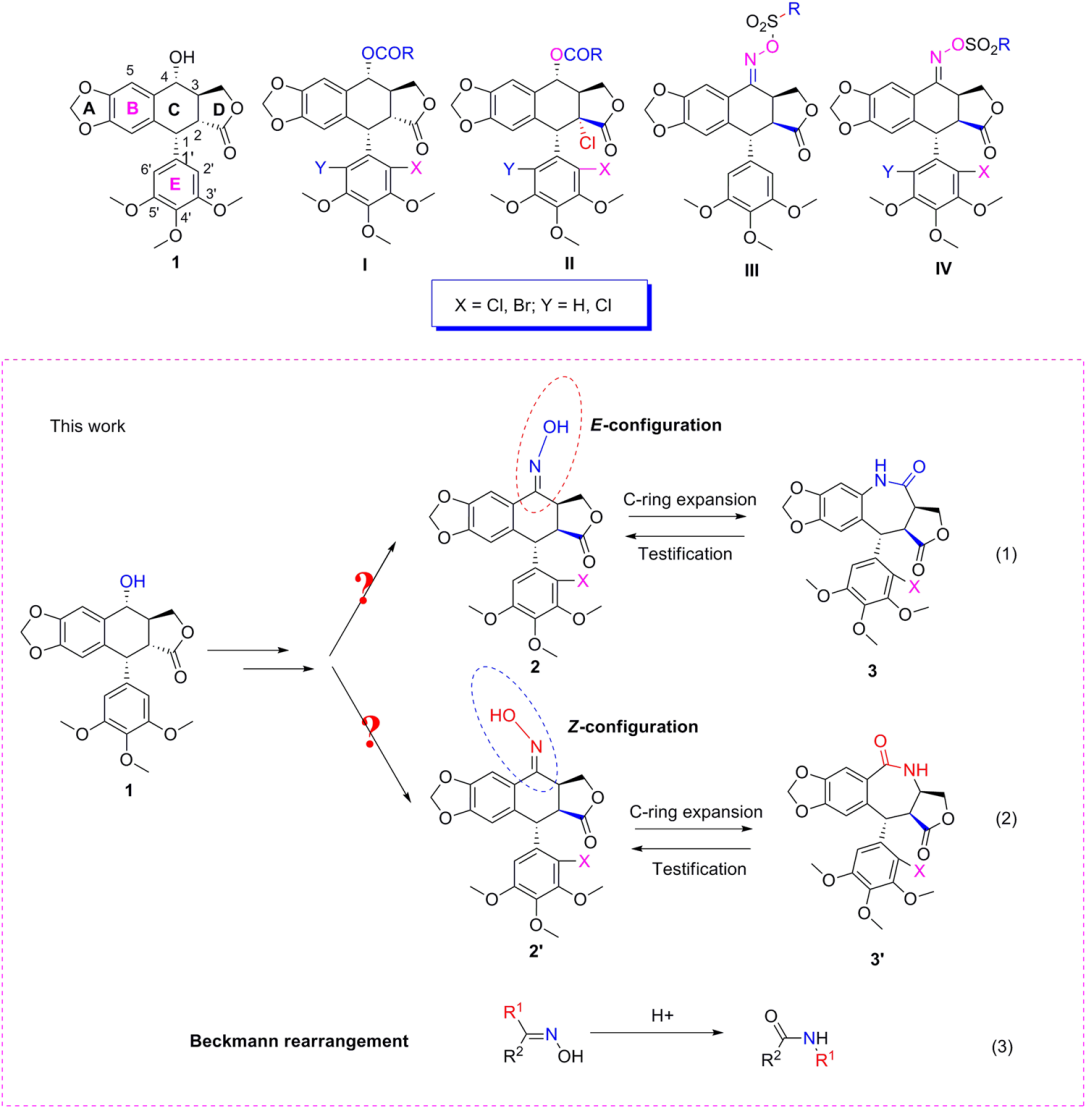
Chemical structures of podophyllotoxin (1) and its derivatives (2, 2′, 3, 3′, and I-IV).

Podophyllotoxin (**1**, Fig. 1), isolated from the roots and rhizomes of *Podophyllum hexandrum*, is a naturally occurring aryltetralin lignan and contains five rings (labeled A–E). In addition to its mesmerizing structure, compound **1** has been used as the lead compound for preparation of potent anticancer drugs (e.g., etoposide, teniposide and etopophos)^9–11^, and insecticidal/antifungal agents^12–14^. More recently, we have prepared a series of 4α-acyloxy-2′(2′,6′)-(di)halogenopodophyllotoxins^14^ (**I**, Fig. 1), 2α-chloro-4α-acyloxy-2′(2′,6′)-(di)halogenopicropodophyllotoxins^7^ (**II**, Fig. 1), and oxime sulfonates of picropodophyllotoxin^15^ (**III**, Fig. 1)/ 2′(2′,6′)-(di)chloropicropodophyllotoxins^16^ (**IV**, Fig. 1) as insecticidal agents, and found some derivatives exhibited more potent insecticidal activity than toosendanin, a commercial botanical insecticide isolated from *Melia azedarach*. Especially it demonstrated that introduction of a chlorine atom at the C-2′ or C-2′,6′ position on the E-ring of picropodophyllotoxin was important for the insecticidal activity^16^. To the best of our knowledge, structural modification on the C-ring expansion of podophyllotoxins has not been carried out. In continuation of our program aimed at the discovery and development of new podophyllotoxin-based insecticidal agents, here we wanted to prepare unusual 7-membered lactam derivatives of podophyllotoxins by C-ring expansion reaction. Additionally, as shown in Fig. 1, the hydroxylamination products, picropodophyllotoxin C4-oximes (**2**, Eq. 1) and (**2**′, Eq. 2), might have *E*- and *Z*- configurations, respectively. Beckmann rearrangement is a well-known organic name reaction and its reaction mechanism has been fully proven^17–19^. According to the Beckmann rearrangement rule (Eq. 3, Fig. 1)^17–19^, the substituent R^1^ at the anti position to the hydroxyl group on the C=N moiety migrates to its nitrogen atom. Therefore, we envisioned that the configuration of the picropodophyllotoxin C4-oximes could also be confirmed by the corresponding C-ring expansion products via the Beckmann rearrangement.

### Materials and Instruments

**Figure 2:**
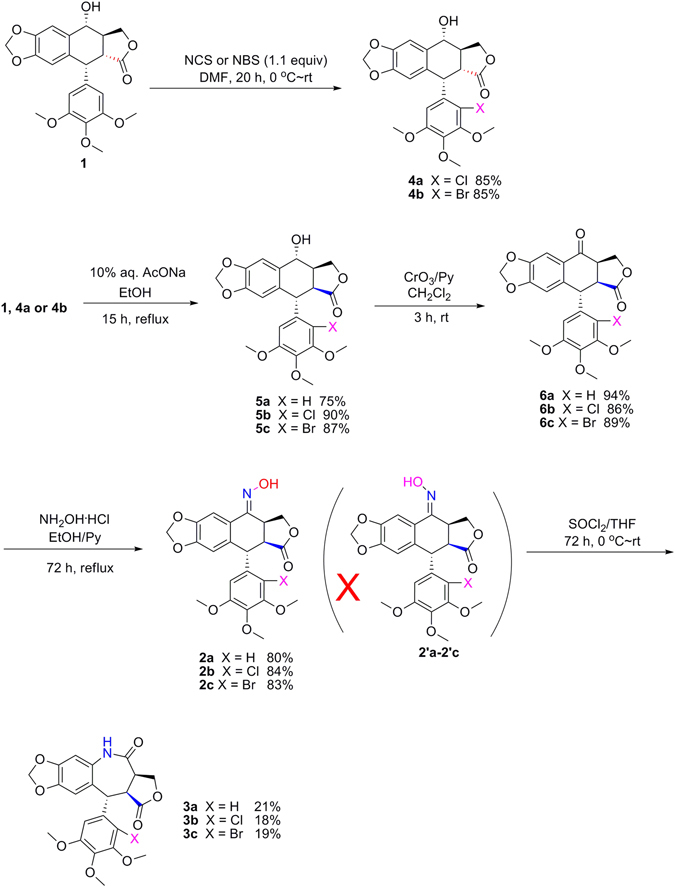
Synthesis of lactam derivatives **3a**–**c** by Beckmann rearrangement.

All chemical reagents were purchased and utilized without further purification. Solvents were used directly or treated with standard methods before use. 2′-Chloropodophyllotoxin (**4a**) and 2′-bromopodophyllotoxin (**4b**) were all prepared in 85% yields (Fig. 2) according to our previous method^14^. Melting points (mp) were determined on a XT-4 digital melting point apparatus (Beijing Tech Instrument Co., Ltd., Beijing, China) and were uncorrected. Optical rotation was measured on a Rudolph Research Analytical Autopol III automatic polarimeter. Proton nuclear magnetic resonance spectra (^1^H NMR) was recorded in CDCl_3_ on a Bruker Avance 500 MHz instrument, and tetramethylsilane (TMS) was used as the internal standard.

### General procedure for synthesis of compounds 5a–5c

A mixture of compound **1**, **4a** or **4b** (1.0 mmol), absolute ethanol (10 mL) and 10% aq. CH_3_CO_2_Na (10 mL) was refluxed, and the reaction process was checked by TLC analysis. After 15 h, the mixture was cooled at 0 °C and filtered to give the solid, which was further recrystallized from absolute ethanol to give **5a**–**5c**.

*Data for* **5a**: Yield = 75%, white solid, m.p. 222–223 °C; [α]^20^_D_ = +5 (*c* 3.2 mg/mL, CHCl_3_); ^1^H NMR (500 MHz, DMSO-*d*_6_) *δ*: 7.06 (s, 1 H, H-5), 6.60 (s, 2 H, H-2′, 6′), 6.00 (s, 1 H, H-8), 5.91–5.95 (m, 3 H, H-1, OCH_2_O), 4.51 (d, *J* = 9.0 Hz, 1 H, H-4), 4.34–4.41 (m, 2 H, H-11), 3.92 (d, *J* = 7.5 Hz, 1 H, H-2), 3.74 (s, 6 H, 3′-OCH_3_, 5′-OCH_3_), 3.69 (s, 3 H, 4′-OCH_3_), 3.40–3.44 (m, 1 H, H-3).

*Data for* **5b**: Yield = 90%, white solid, m.p. 116–117 °C; [α]^20^_D_ = +5 (*c* 3.2 mg/mL, acetone); ^1^H NMR (500 MHz, CDCl_3_) *δ*: 7.09 (s, 1 H, H-5), 6.61 (s, 1 H, H-8), 6.18 (s, 1 H, H-6′), 5.94 (s, 2 H, OCH_2_O), 4.66 (d, *J* = 9.5 Hz, 1 H, H-1), 4.47 (d, *J* = 9.5 Hz, 1 H, H-4), 4.40–4.43 (m, 1 H, H-11), 4.38 (d, *J* = 5.0 Hz, 1 H, H-11), 3.92 (s, 3 H, 3′-OCH_3_), 3.91 (s, 3 H, 5′-OCH_3_), 3.80 (s, 3 H, 4′-OCH_3_), 3.27–3.30 (m, 1 H, H-2), 2.90 (s, 1 H, 4-OH), 2.60–2.65 (m, 1 H, H-3).

*Data for* **5c**: Yield = 83%, white solid, m.p. 82–84 °C; [α]^20^_D_ = −15 (*c* 3.4 mg/mL, CHCl_3_); ^1^H NMR (500 MHz, CDCl_3_) *δ*: 7.08 (s, 1 H, H-5), 6.61 (s, 1 H, H-8), 6.21 (s, 1 H, H-6′), 5.93 (dd, *J* = 12.5, 1.0 Hz, 2 H, OCH_2_O), 4.65 (d, *J* = 9.5 Hz, 1 H, H-1), 4.51 (s, 1 H, H-4), 4.41-4.47 (m, 2 H, H-11), 3.92 (s, 3 H, 3′-OCH_3_), 3.91 (s, 3 H, 5′-OCH_3_), 3.80 (s, 3 H, 4′-OCH_3_), 3.30 (dd, *J* = 9.0, 6.0 Hz, 1 H, H-2), 2.63–2.67 (m, 1 H, H-3).

### General procedure for synthesis of compounds 6a–6c

A mixture of **5a**, **5b** or **5c** (0.5 mmol), chromium trioxide (CrO_3_, 2.5 mmol), and pyridine (5 mmol) in dry dichloromethane (15 mL) was stirred at room temperature. When the reaction was complete after 3 h, checked by TLC analysis, the mixture was diluted by dichloromethane, washed by saturated aq. NaHSO_3_ and brine, dried over anhydrous Na_2_SO_4_, concentrated under reduced pressure, and purified by silica gel column chromatography to afford **6a**–**6c**.

*Data for* **6a**: Yield = 94%, white solid, m.p. 152–154 °C; [α]^20^_D_ = −164 (*c* 3.4 mg/mL, CHCl_3_); ^1^H NMR (500 MHz, CDCl_3_) *δ*: 7.49 (s, 1 H, H-5), 6.68 (s, 1 H, H-8), 6.23 (s, 2 H, H-2′, 6′), 6.04 (d, *J* = 3.5 Hz, 2 H, OCH_2_O), 4.75 (d, *J* = 9.0 Hz, 1 H, H-11), 4.69 (s, 1 H, H-1), 4.33-4.36 (m, 1 H, H-11), 3.80 (s, 3 H, 4′-OCH_3_), 3.75 (s, 6 H, 3′, 5′-OCH_3_), 3.30–3.32 (m, 2 H, H-2, 3).

*Data for* **6b**: Yield = 86%, white solid, m.p. 105–107 °C; [α]^20^_D_ = −30 (*c* 3.4 mg/mL, acetone); ^1^H NMR (500 MHz, CDCl_3_) *δ*: 7.51 (s, 1 H, H-5), 6.68 (s, 1 H, H-8), 6.07 (d, *J* = 1.0 Hz, 2 H, OCH_2_O), 5.85 (s, 1 H, H-6′), 5.21 (d, *J* = 1.5 Hz, 1 H, H-1), 4.75 (d, *J* = 9.0 Hz, 1 H, H-11), 4.32–4.35 (m, 1 H, H-11), 3.95 (s, 3 H, 3′-OCH_3_), 3.85 (s, 3 H, 5′-OCH_3_), 3.54 (s, 3 H, 4′-OCH_3_), 3.42 (dd, *J* = 8.0, 2.0 Hz, 1 H, H-2), 3.15–3.18 (m, 1 H, H-3).

*Data for* **6c**: Yield = 80%, white solid, m.p. 98–100 °C; [α]^20^_D_ = −53 (*c* 3.2 mg/mL, CHCl_3_); ^1^H NMR (500 MHz, CDCl_3_) *δ*: 7.51 (s, 1 H, H-5), 6.67 (s, 1 H, H-8), 6.06 (d, *J* = 8.5 Hz, 2 H, OCH_2_O), 5.88 (s, 1 H, H-6′), 5.22 (s, 1 H, H-1), 4.74 (d, *J* = 9.5 Hz, 1 H, H-11), 4.32–4.35 (m, 1 H, H-11), 3.93 (s, 3 H, 3′-OCH_3_), 3.84 (s, 3 H, 5′-OCH_3_), 3.53 (s, 3 H, 4′-OCH_3_), 3.43 (dd, *J* = 8.0, 1.5 Hz, 1 H, H-2), 3.14–3.16 (m, 1 H, H-3).

### General procedure for synthesis of compounds 2a–2c

A mixture of **6a**, **6b** or **6c** (0.53 mmol), hydroxylamine hydrochloride (0.8 mmol), and pyridine (2.12 mmol) in absolute ethanol (20 mL) was refluxed, and the reaction process was checked by TLC analysis. After 72 h, the solvent was removed under reduced pressure and saturated aq. NaHCO_3_ was added to the residue, which was extracted with ethyl acetate. The combined organic phase was dried over anhydrous Na_2_SO_4_, filtered, concentrated under reduced pressure, and purified by silica gel column chromatography to afford **2a**–**2c**.

*Data for* **2a**: Yield = 80%, White solid, m.p. 114–116 °C; [α]^20^_D_ = −53 (*c* 3.0 mg/mL, CHCl_3_); ^1^H NMR (500 MHz, CDCl_3_) *δ*: 7.27 (s, 1 H, H-5), 6.69 (s, 1 H, H-8), 6.25 (s, 2 H, H-2′, 6′), 5.98 (d, *J* = 2.5 Hz, 2 H, OCH_2_O), 4.57 (d, *J* = 2.0 Hz, 1 H, H-1), 4.51–4.52 (m, 2 H, H-11), 3.98–4.02 (m, 1 H, H-3), 3.79 (s, 3 H, 4′-OCH_3_), 3.74 (s, 6 H, 3′, 5′-OCH_3_), 3.24 (dd, *J* = 2.5, 8.5 Hz, 1 H, H-2).

*Data for* **2b**: Yield = 83%, white solid, m.p. 106–107 °C; [α]^20^_D_ = −27 (*c* 3.4 mg/mL, acetone); ^1^H NMR (500 MHz, CDCl_3_) *δ*: 7.27 (s, 1 H, H-5), 6.70 (s, 1 H, H-8), 5.99 (s, 2 H, OCH_2_O), 5.95 (s, 1 H, H-6′), 5.07 (d, *J* = 2.0 Hz, 1 H, H-1), 4.47–4.53 (m, 2 H, H-11), 3.93 (s, 3 H, 3′-OCH_3_), 3.83–3.86 (m, 4 H, H-3, 5′-OCH_3_), 3.52 (s, 3 H, 4′-OCH_3_), 3.43 (dd, *J* = 8.5, 2.5 Hz, 1 H, H-2).

*Data for* **2c**: Yield = 82%, white solid, m.p. 124–126 °C; [α]^20^_D_ = −32 (*c* 3.9 mg/mL, CHCl_3_); ^1^H NMR (500 MHz, CDCl_3_) *δ*: 7.28 (s, 1 H, H-5), 6.69 (s, 1 H, H-8), 6.00 (s, 1 H, H-6′), 5.99 (s, 2 H, OCH_2_O), 5.07 (d, *J* = 2.5 Hz, 1 H, H-1), 4.46–4.52 (m, 2 H, H-11), 3.91 (s, 3 H, 3′-OCH_3_), 3.83–3.86 (m, 4 H, H-3, 5′-OCH_3_), 3.54 (s, 3 H, 4′-OCH_3_), 3.43 (d, *J* = 8.5 Hz, 1 H, H-2).

### General procedure for synthesis of compounds 3a–3c

A solution of thionyl chloride (0.63 mL) in tetrahydrofuran (5 mL) was added dropwise to a solution of **2a**, **2b** or **2c** (0.25 mmol) in tetrahydrofuran (5 mL) at 0 °C. Then the above mixture was stirred, and the reaction temperature was gradually raised to room temperature. The reaction process was checked by TLC analysis. After 72 h, water was added to the mixture, and its pH value was adjusted with ammonia to 8-9. Subsequently, the mixture was extracted with dichloromethane. The combined organic phase was washed by water, 5% aq. NaHCO_3_ and brine, dried over anhydrous Na_2_SO_4_, concentrated under reduced pressure, and purified by preparative thin-layer chromatography (PTLC) to produce **3a**–**3c**.

*Data for* **3a**: Yield = 21%, white solid, m.p. 232–234 °C; [α]^20^_D_ = −85 (*c* 3.1 mg/mL, CHCl_3_); IR cm^−1^ (KBr): 3443, 2930, 2844, 1767, 1680, 1594, 1493, 1474, 1240, 1130, 1039; ^1^H NMR (500 MHz, CDCl_3_) *δ*: 8.29 (s, 1 H, NH), 6.56 (s, 1 H), 6.47 (brs, 2 H), 6.29 (s, 1 H), 5.94 (dd, *J* = 8.0, 1.0 Hz, 2 H, OCH_2_O), 4.99 (brs, 1 H, H-1), 4.37 (d, *J* = 12.0 Hz, 1 H, H-11), 4.21 (dd, *J* = 8.5, 6.0 Hz, 1 H, H-2), 3.87 (s, 4 H, OCH_3_, H-11), 3.83 (s, 6 H, 2 × OCH_3_), 3.38 (brs, 1 H, H-3); HRMS (ESI): Calcd for C_22_H_22_O_8_N ([M + H]^+^), 428.1340; Found, 428.1344.

*Data for* **3b**: Yield = 18%, white solid, m.p. 155–157 °C; [α]^20^_D_ = −141 (*c* 2.1 mg/mL, CHCl_3_); ^1^H NMR (500 MHz, CDCl_3_) *δ*: 7.57 (s, 1 H), 6.69 (s, 1 H), 6.57 (s, 1 H), 6.09 (brs, 1 H, H-1), 5.94 (d, *J* = 6.0 Hz, 2 H, OCH_2_O), 5.11 (d, *J* = 9.0 Hz, 1 H, H-11), 4.83 (d, *J* = 13.0 Hz, 1 H, H-11), 4.21 (dd, *J* = 9.5, 5.5 Hz, 1 H, H-2), 3.94 (s, 3 H, 3′-OCH_3_), 3.90 (s, 3 H, 4′-OCH_3_), 3.89 (s, 3 H, 5′-OCH_3_), 3.35 (dd, *J* = 9.5, 5.5 Hz, 1 H, H-3); HRMS (ESI): Calcd for C_22_H_21_O_8_NCl ([M + H]^+^), 462.0950; Found, 462.0949.

*Data for* **3c**: Yield = 19%, white solid, m.p. 158–160 °C; [α]^20^_D_ = −116 (*c* 2.9 mg/mL, CHCl_3_); ^1^H NMR (500 MHz, CDCl_3_) *δ*: 7.68 (s, 1 H), 6.72 (s, 1 H), 6.57 (s, 1 H), 6.06 (s, 1 H, H-1), 5.94 (dd, *J* = 5.5, 1.0 Hz, 2 H, OCH_2_O), 5.11 (d, *J* = 9.5 Hz, 1 H, H-11), 4.84 (d, *J* = 13.0 Hz, 1 H, H-11), 4.20 (dd, *J* = 9.0, 5.5 Hz, 1 H, H-2), 3.94 (s, 3 H, 3′-OCH_3_), 3.90 (s, 3 H, 4′-OCH_3_), 3.89 (s, 3 H, 5′-OCH_3_), 3.34 (dd, *J* = 9.5, 5.5 Hz, 1 H, H-3); ^13^C NMR (125 MHz, CDCl_3_) *δ*: 173.0, 169.8, 152.5, 151.6, 147.1, 146.1, 142.5, 132.1, 129.4, 128.9, 112.8, 108.2, 107.6, 104.1, 101.7, 65.4, 61.1, 61.0, 56.4, 49.9, 42.9, 29.7; HRMS (ESI): Calcd for C_22_H_21_O_8_NBr ([M + H]^+^), 506.0445; Found, 506.0441.

### Biological assay

The insecticidal activity of **1**–**6** was tested as the mortality rate values by using the leaf-dipping method^14^, against the pre-third-instar larvae of oriental armyworm, *Mythimna separata* (Walker). Toosendanin, isolated from *Melia azedarach*, was used as a positive control and supplied by Research & Development Center of Biorational Pesticide, Northwest A&F University, Shaanxi province, China. For each compound, 30 larvae (10 larvae per group) were used. Acetone solutions of all tested compounds, and toosendanin were prepared at the concentration of 1 mg/mL. Fresh wheat leaves were dipped into the corresponding solution for 3 s, then taken out, and dried in a room. Leaves treated with acetone alone were used as a blank control group. Several treated leaves were kept in each dish, where every 10 larvae were raised. If the treated leaves were consumed, additional treated leaves were added to the dish. After 48 h, compound-soaked leaves were removed, and untreated fresh ones were added to all dishes till adult emergence. The experiment was carried out in a conditioned room (25 ± 2 °C, 65–80% relative humidity (RH), 12 h/12 h (light/dark) photoperiod). The insecticidal activity of the tested compounds against *M. separata* was calculated by the formula

$${\rm{corrected}}\,{\rm{mortality}}\,{\rm{rate}}( \% )=(T-C)\times 100/(100 \% -C)$$

Where *T* is the mortality rate in the group treated with the tested compounds, and *C* is the mortality rate in the blank control group (*T* and *C* were all expressed as the percentage).

## Results and Discussion

**Figure 3:**
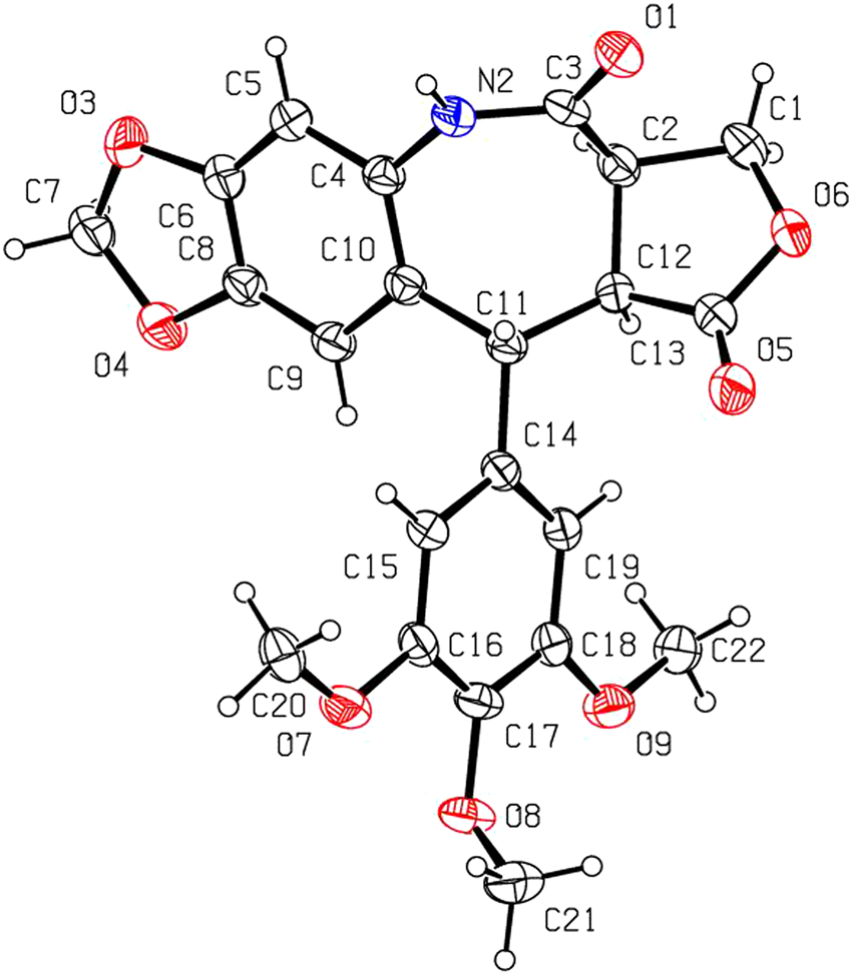
The X-ray crystal structure of **3a**. Drawing by Hui Xu.

**Figure 4:**
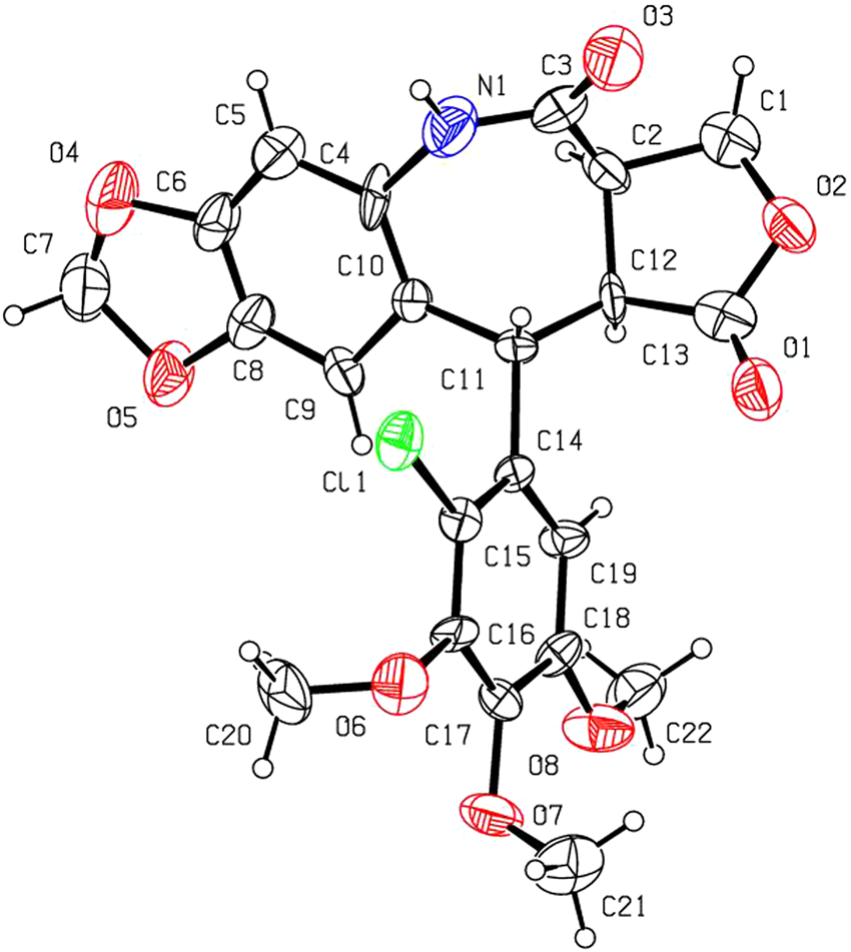
The X-ray crystal structure of **3b**. Drawing by Hui Xu.

**Figure 5:**
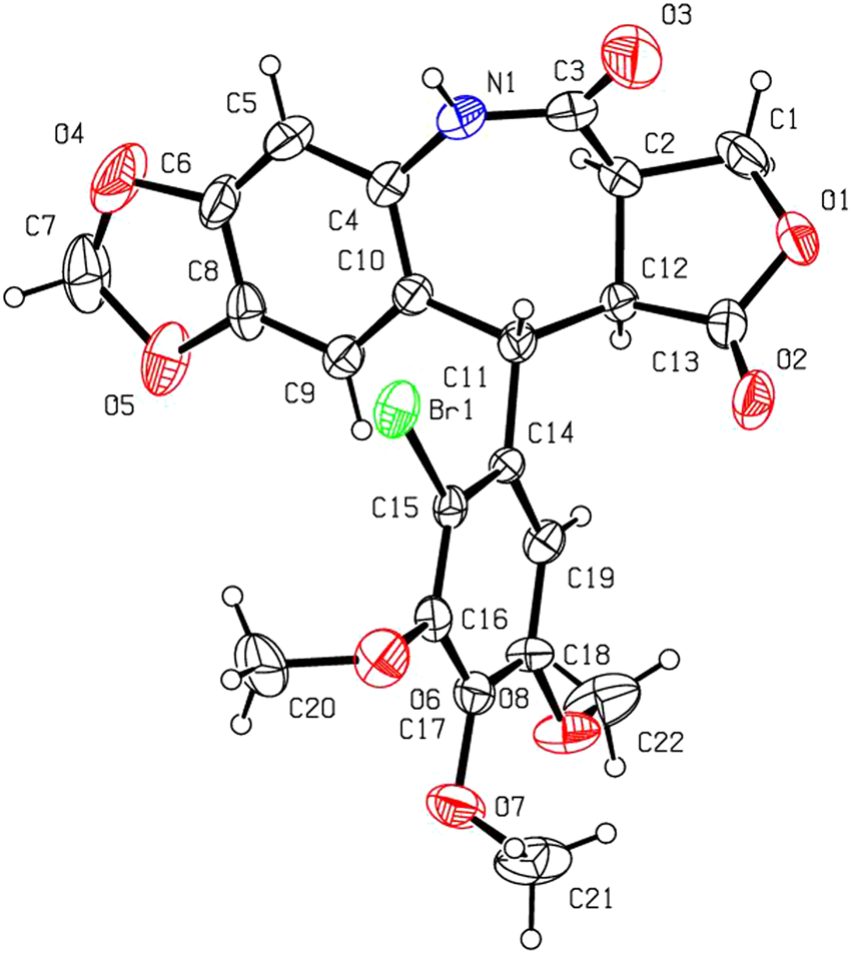
The X-ray crystal structure of **3c**. Drawing by Hui Xu.

As shown in Fig. 2, 2′-chloropodophyllotoxin (**4a**) and 2′-bromopodophyllotoxin (**4b**) were smoothly prepared by reaction of **1** with 1.1 equiv of *N*-chlorosuccinimide (NCS) and *N*-bromosuccinimide (NBS), respectively^14^. Then, picropodophyllotoxin (**5a**), 2′-chloropicropodophyllotoxin (**5b**) and 2′-bromopicropodophyllotoxin (**5c**) were obtained by reaction of 10% aq. NaOAc with **1**, **4a** and **4b**, respectively^15, 16^. Subsequently, oxidation of **5a–c** in the presence of chromium trioxide (CrO_3_) and pyridine afforded picropodophyllones (**6a–c**)^15, 16^. When **6a–c** further reacted with hydroxylamine hydrochloride, only picropodophyllotoxin C4-oximes **2a–c** (*E* configuration), testified in our previous paper^15, 16^, were selectively obtained, whereas their *Z* configuration isomers **2**′**a–c** were not detected. Finally, in the presence of thionyl chloride, three novel 7-membered lactam derivatives of podophyllotoxin (**3a–c**) were produced via Beckmann rearrangement of the C-ring of **2a–c**. The steric configurations of **3a–c** were unambiguously identified by X-ray crystallography (Figs 3–5). It clearly demonstrated that the NH groups of 7-membered lactams of **3a–c** were connected with their B-ring (phenyl ring). Based on the Beckmann rearrangement rule^17–19^, the substituent at the anti position to the hydroxyl group on the C=N moiety migrates to its nitrogen atom. Therefore, the phenyl ring should be at the anti position to the hydroxyl group, and it further demonstrated that picropodophyllotoxin C4-(*E*) oximes **2a–c** was selectively produced. Three crystallographic data (excluding structure factors) for the structures of **3a–c**, in this paper have been deposited with the Cambridge Crystallographic Data Centre as supplementary publication number CCDC 1495773, 1495719, and 1495720, respectively. Copies of the data can be obtained, free of charge, on application to CCDC, 12 Union Road, Cambridge CB2 1EZ, UK [fax: +44 (0)1223 336033 or e-mail: deposit@ccdc.cam.ac.uk].

**Figure 6:**
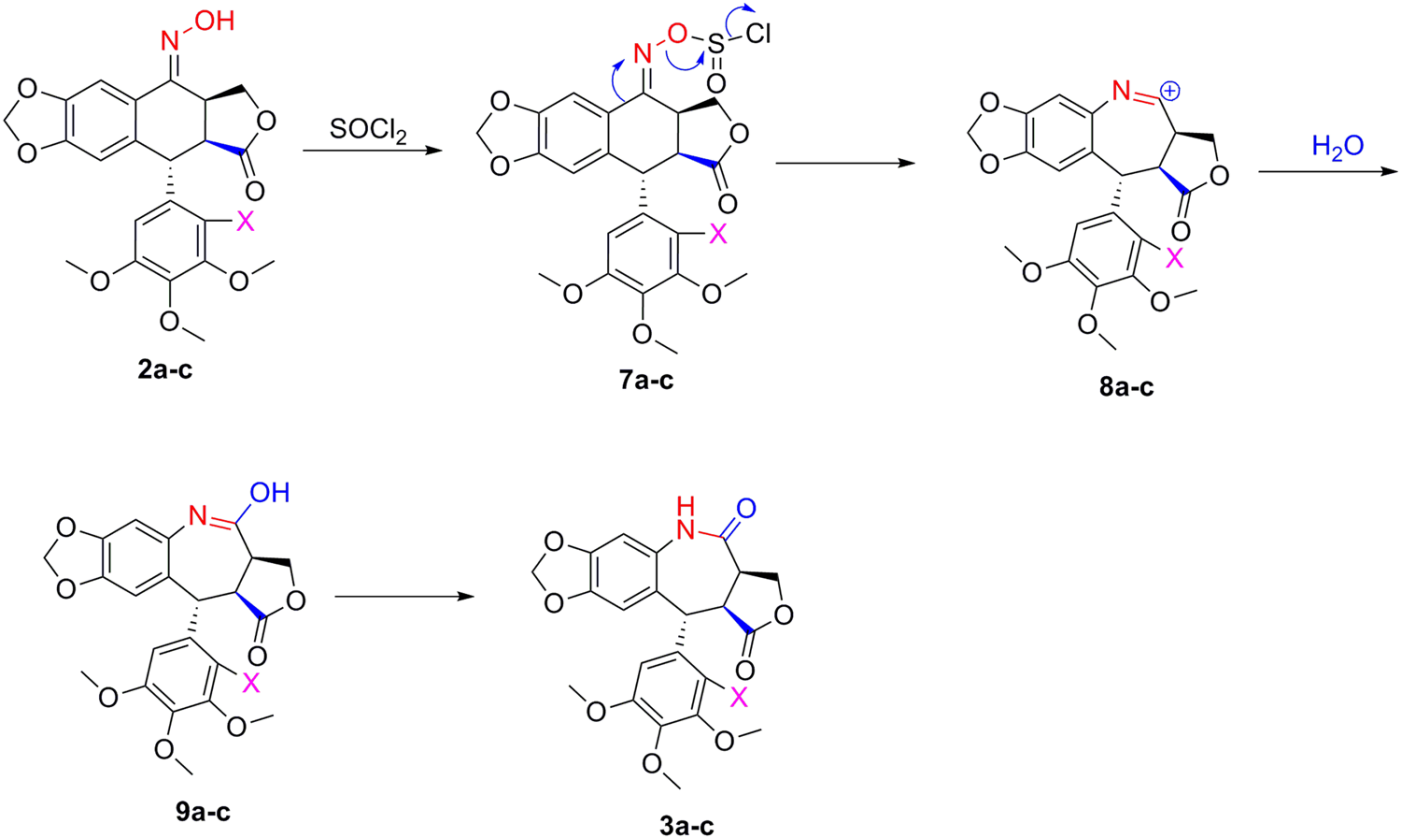
Mechanism for synthesis of 7-membered lactams 3**a**–**c** by thionyl chloride-mediated Beckmann rearrangement.

Moreover, according to the Beckmann rearrangement rule^17–19^, the mechanism for synthesis of **3a**–**c** by thionyl chloride-mediated Beckmann rearrangement was described in Fig. 6. First, thionyl chloride activated the oxime fragments of **2a–c** to give **7a**–**c**. Then the phenyl (B-ring) at an anti position to the hydroxyl group migrated to the oxime nitrogen atom of **7a**–**c**, and the corresponding key nitrilium cations **8a**–**c** were produced. Finally, compounds **8a**–**c** were further hydrolyzed to **3a**–**c** via the intermediates **9a**–**c**.

As shown in Table 1, the insecticidal activity of compounds **1-6** against the pre-third-instar larvae of *M. separata in vivo* was tested by the leaf-dipping method at a concentration of 1 mg/mL. Toosendanin, a commercial botanical insecticide isolated from *Melia azedarach*, was used as the positive control at 1 mg/mL. Leaves treated with acetone alone were used as a blank control group. The corresponding mortality rates of the treated groups after 35 days were higher than those after 10 and 20 days in the same as in our previous papers^14–16^. For example, the corrected mortality rates of **3b** against *M. separata* after 10 and 20 days were 10% and 16.7%, respectively. However, it was remarkablely increased to 56.7% after 35 days, which was more than 5-fold of that after 10 days. Among all derivatives, two lactams **3b** and **3c** exhibited more potent insecticidal activity than the positive control toosendanin. For example, the final mortality rates (FMRs) of **3b** and **3c** were 56.7% and 60.0%, respectively; whereas the FMRs of the precursor **1** and toosendanin were 33.3% and 50.0%, respectively. It further demonstrated that introduction of a halogen atom at the C-2′ position on the E-ring of picropodophyllotoxin or podophyllotoxin was important for the insecticidal activity^14, 16^. For example, the FMRs of **2b** and **2c** were 33.3% and 43.3%, respectively; whereas the FMRs of **2a** was 23.3%; the FMRs of **4a** and **4b** were 50.0% and 46.7%, respectively; whereas the FMRs of **1** was 33.3%; the FMRs of **5b** and **5c** were 50.0% and 43.3%, respectively; whereas the FMRs of **5a** was 36.7%; the FMRs of **6b** and **6c** were 36.7% and 46.7%, respectively; whereas the FMRs of **6a** was 30.0%. Finally, to obtain the EC_50_ value, further biological assay for compound **3c** was conducted; the EC_50_ value of compound **3c** was 0.809 mg/mL (See Supporting Information).

**Table 1:** Insecticidal Activity of compounds 1–6 against *M. separata* on Leaves Treated with a Concentration of 1 mg/mL.

Compound	Corrected mortality rate (%)^a^		
	10 days	20 days	35 days
**1**	0 ± 0	13.3 ± 3.3	33.3 ± 3.3
**2a**	3.3 ± 3.3	13.3 ± 3.3	23.3 ± 3.3
**2b**	10.0 ± 0	20.0 ± 5.8	33.3 ± 3.3
**2c**	6.7 ± 3.3	16.7 ± 3.3	43.3 ± 3.3
**3a**	10.0 ± 0	26.7 ± 3.3	43.3 ± 0
**3b**	10.0 ± 0	16.7 ± 3.3	56.7 ± 3.3
**3c**	6.7 ± 3.3	16.7 ± 3.3	60.0 ± 3.3
**4a**	10.0 ± 0	33.3 ± 3.3	50.0 ± 5.8
**4b**	3.3 ± 3.3	20.0 ± 0	46.7 ± 3.3
**5a**	10.0 ± 0	16.7 ± 3.3	36.7 ± 3.3
**5b**	16.7 ± 3.3	33.3 ± 3.3	50.0 ± 0
**5c**	6.7 ± 3.3	23.3 ± 3.3	43.3 ± 3.3
**6a**	16.7 ± 3.3	23.3 ± 3.3	30.0 ± 5.8
**6b**	13.3 ± 3.3	20.0 ± 5.8	36.7 ± 3.3
**6c**	6.7 ± 3.3	20.0 ± 0	46.7 ± 3.3
toosendanin	3.3 ± 3.3	26.7 ± 3.3	50.0 ± 0

^a^Values are means ± S.D. of three replicates.

## Conclusion

In summary, three novel and unusual 7-membered lactam derivatives of podophyllotoxin were obtained by thionyl chloride-mediated ring-expanded Beckmann rearrangement of picropodophyllotoxin C4-oximes. The steric configurations of **3a–c** were all unambiguously confirmed by X-ray crystallography. It further demonstrated that when picropodophyllones reacted with hydroxylamine hydrochloride, only picropodophyllotoxin C4-(*E*) oximes were selectively produced. Especially compounds **3b** and **3c** showed more potent insecticidal activity than toosendanin against *M. separata*. It showed that introducing a halogen atom at the C-2′ position on the E-ring of picropodophyllotoxin/podophyllotoxin was important for the insecticidal activity. It will pave the way for further design and structural modifications of podophyllotoxin as botanical insecticidal agents.

## Electronic supplementary material

supporting information
