# Supplementary material for: Seven-Membered Lactam Derivatives of Podophyllotoxins as New Pesticidal Agents
Source: Sci Rep. 2017 Jun 20;7:3917. doi: 10.1038/s41598-017-04136-3 (PMC5478614; doi:10.1038/s41598-017-04136-3)

**Seven-Membered Lactam Derivatives of Podophyllotoxins as New Pesticidal Agents**

Xiaoyan Zhi^1,2,*^, Yuanyuan Zhang^1,*^, Jiulin Huang^1^ & Hui Xu^1,3^

^1^Research Institute of Pesticidal Design & Synthesis, College of Chemistry and Pharmacy, Northwest A&F University, Yangling 712100, Shaanxi Province, P. R. China.

^2^College of Agriculture, Shanxi Agriculture University, Taigu 030801, Shanxi Province, P. R. China.

^3^Shaanxi Key Laboratory of Natural Products & Chemical Biology, and College of Plant Protection, Northwest A&F University, Yangling 712100, Shaanxi Province, P. R. China.

^*^These authors contributed equally to this work.

Correspondence and requests for materials should be addressed to H.X. ([orgxuhui@nwsuaf.edu.cn](mailto:orgxuhui@nwsuaf.edu.cn)); Telephone: +86(0)29-87091952; Fax: +86(0)29-87091952.

**1. The EC_50_ value of compound 3c.**

**Table S1.** Corrected mortality rates at different five concentrations of compound **3c** against *Mythimna separata.*

| Concentration（mg/mL） | Corrected mortality rate (%) |
| --- | --- |
| 1.2 | 62.96 |
| 0.6 | 40.74 |
| 0.3 | 22.22 |
| 0.15 | 14.81 |
| 0.075 | 7.41 |


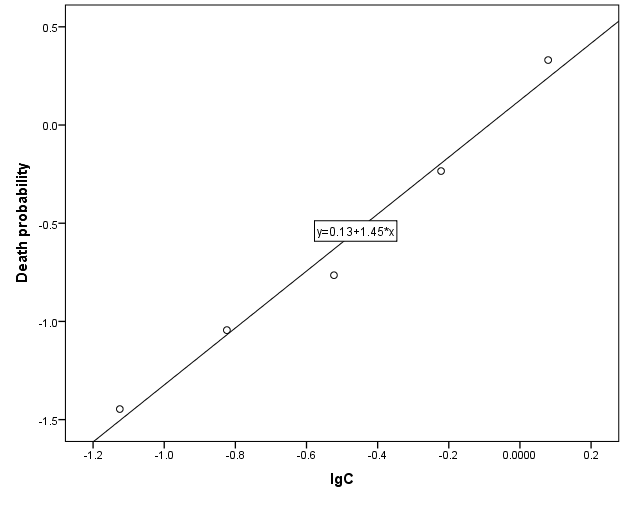


**Figure S1.** A decent dose-response curve for **3c**.

**Table S2.** The EC_50_ value result of compound **3c.**

| Compd. | EC_50_^a^  (mg/mL) | 95% Confidence interval | [Toxic](http://cn.bing.com/dict/search?q=Toxic&FORM=BDVSP6&mkt=zh-cn) [regression](http://cn.bing.com/dict/search?q=regression&FORM=BDVSP6&mkt=zh-cn)  [equation](http://cn.bing.com/dict/search?q=equation&FORM=BDVSP6&mkt=zh-cn) | [Correlation](http://cn.bing.com/dict/search?q=correlation&FORM=BDVSP6&mkt=zh-cn) [index](http://cn.bing.com/dict/search?q=index&FORM=BDVSP6&mkt=zh-cn) |
| --- | --- | --- | --- | --- |
| **3c** | 0.809 | 0.648-1.083 | y=0.13+1.45x | 0.991 |

^a^SPSS 23.0

**2. For compounds 5a,b; 6a,b; and 2a,b were reported in our previous paper (Wang, et al., *J. Agric. Food Chem.* 2015, 63, 6668–6674), the NMR spectra of 5c, 6c, 2c, and 3a-c were supplied.**


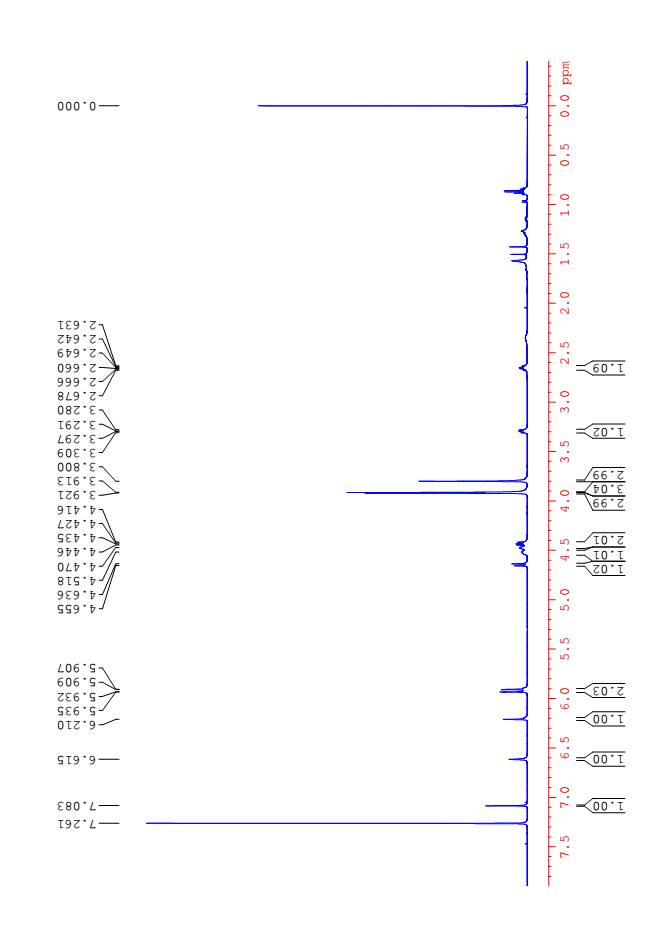


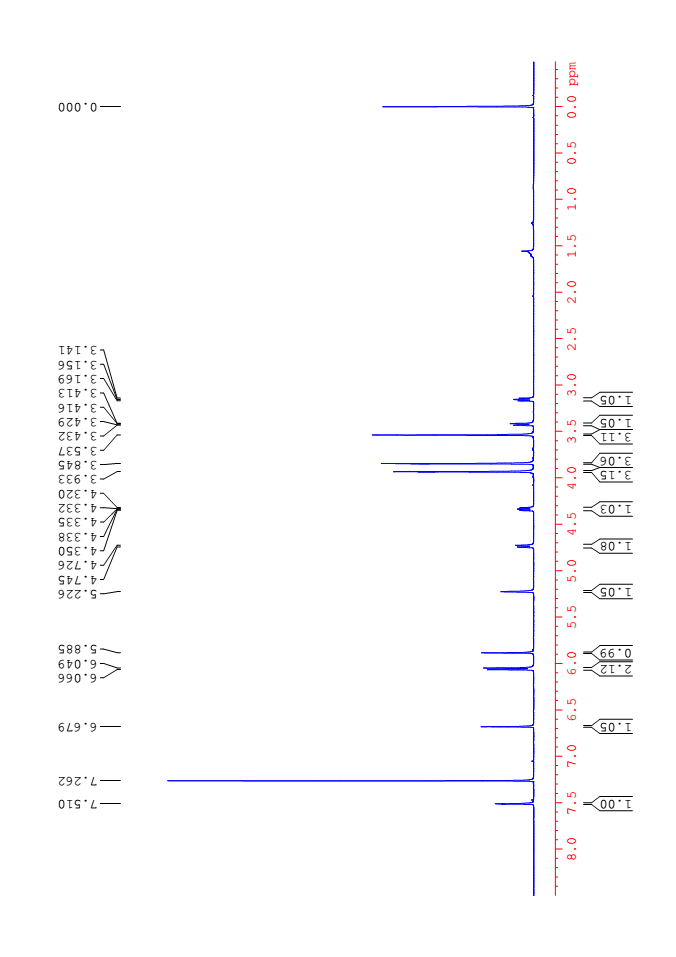


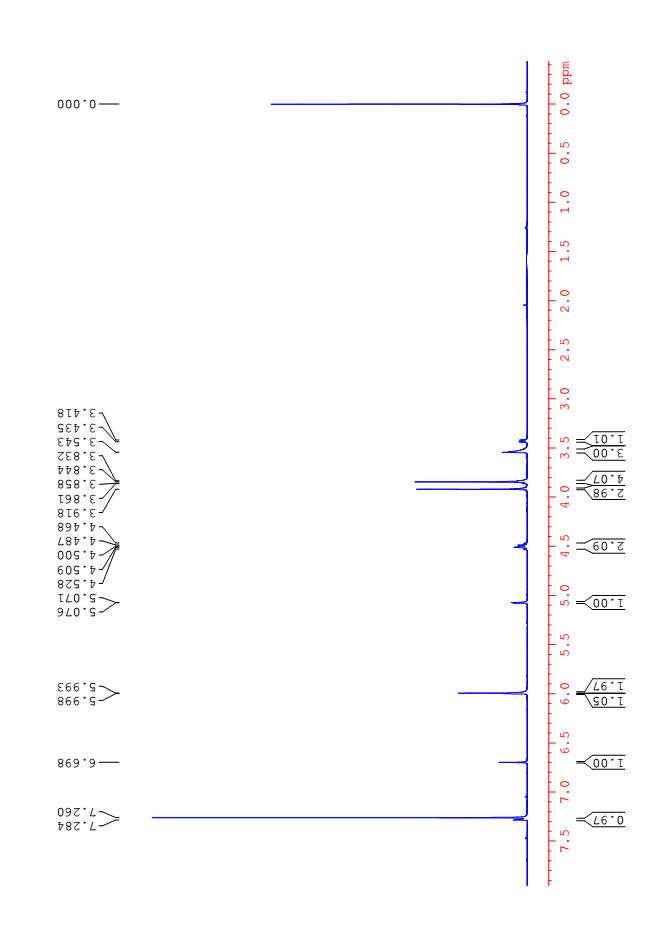


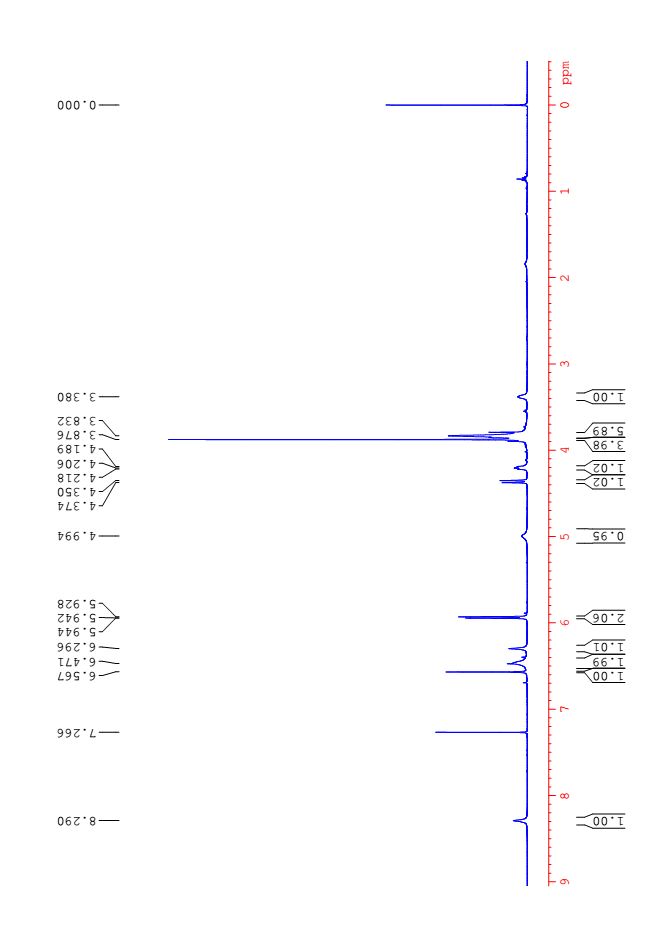


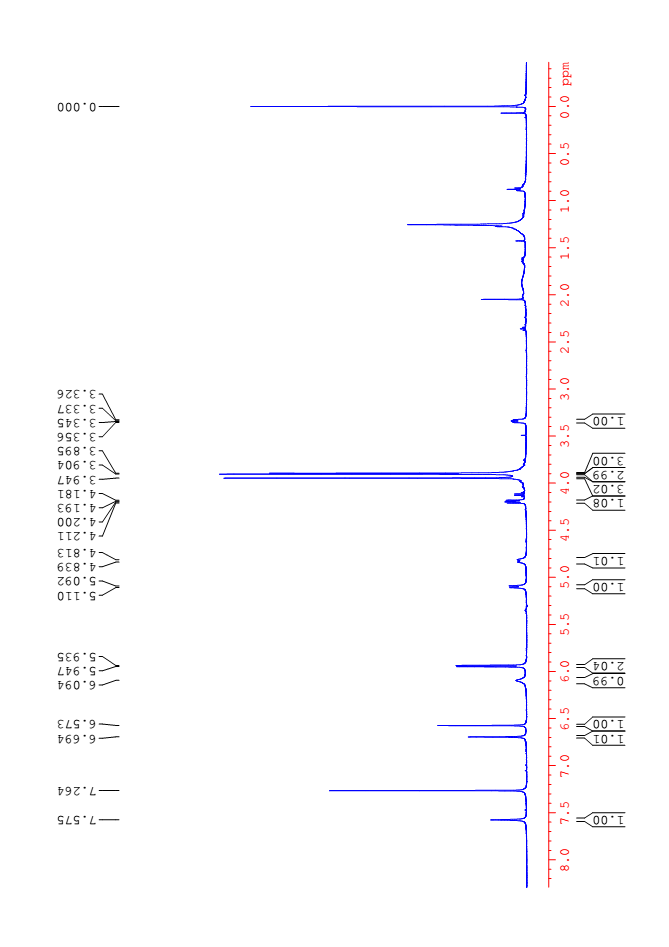


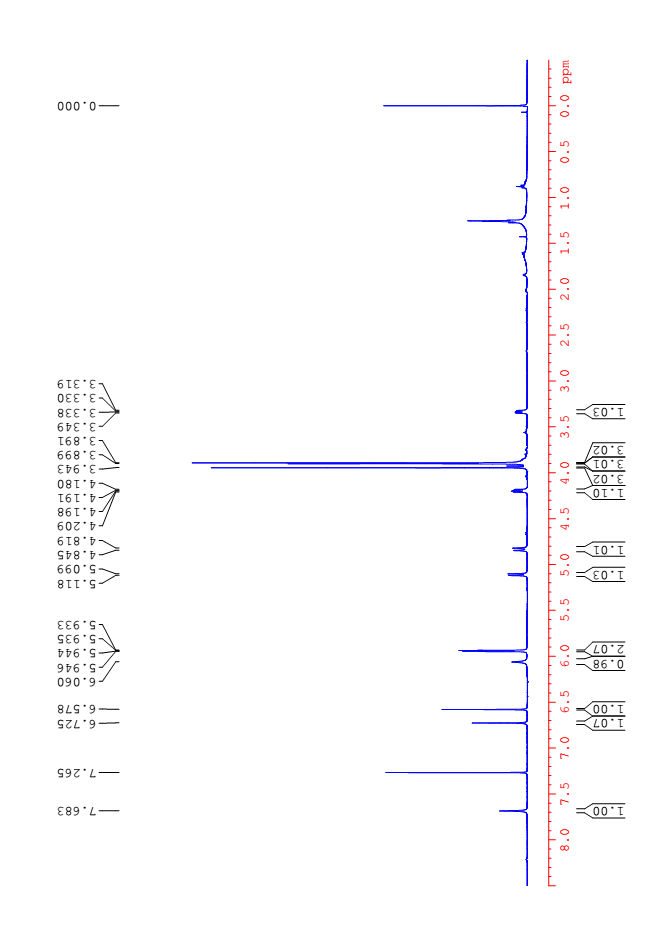


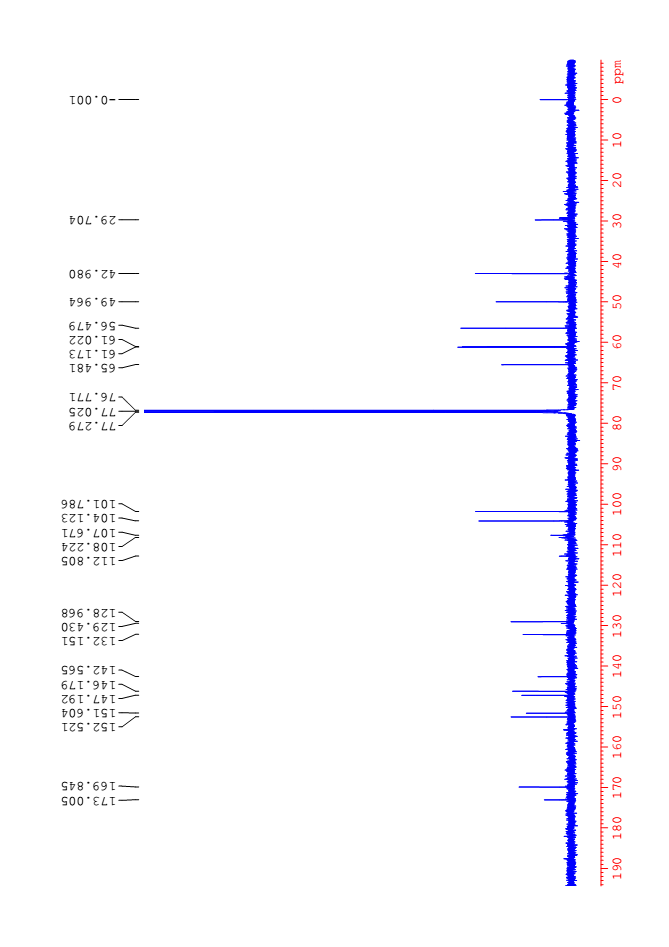

Supplement: Supplementary file 1 — supporting information [file 41598_2017_4136_MOESM1_ESM.doc]
